# Supplementary material for: Influences of Cluster Thinning on Fatty Acids and Green Leaf Volatiles in the Production of Cabernet Sauvignon Grapes and Wines in the Northwest of China
Source: Plants (Basel). 2024 Apr 28;13(9):1225. doi: 10.3390/plants13091225 (PMC11085434; doi:10.3390/plants13091225)
Supplement: Supplementary file 1 [file plants-13-01225-s001.zip › plants-2954624-supplementary.pdf]

Supplementary materials for:

Influences of Cluster Thinning on Fatty Acids and Green Leaf Volatiles in the Production of Cabernet Sauvignon Grapes and Wines in the Northwest of China

Xiaoyu Xu [1,2,†], Chifang Cheng [3,†], Xu Qian [4], Ying Shi [1,2], Changqing Duan [1,2], Yibin Lan [1,2,*]

[1] Centre for Viticulture & Enology, College of Food Science and Nutritional Engineering, China Agricultural University, Beijing 100083, China; xuxiaoyu62429@163.com (X.X.); shiy@cau.edu.cn (Y.S.); chqduan@cau.edu.cn (C.D.)

[2] Key Laboratory of Viticulture and Enology, Ministry of Agriculture and Rural Affairs, College of Food Science and Nutritional Engineering, China Agricultural University, Beijing 100083, China

[3] Xinjiang Wine Industry Innovation Research Institute, Manasi 832200, China; chengchifang@citicguoanwine.com

[4] School of Biology and Food Engineering, Changshu Institute of Technology, Changshu 215500, China; qianxu@cslg.edu.cn

* Correspondence: lanyibin@cau.edu.cn; Tel.: +86-010-62-738-658

† These authors contributed equally to this work.

Catalog

Supplementary figures **1**

Figure S1. Effects of cluster thinning treatments on berry weight, total soluble solids (TSS) content and titratable acidity (TA) in berries of Cabernet Sauvignon during grape development in two seasons (2011-2012). CT-1, one cluster per shoot thinned, 1 cluster/shoot reserved; (B) CT-2, one cluster every other shoot thinned, average 1.5 clusters/shoot reserved; (C) CT-3, not thinned, 2 clusters/shoot reserved. 1

Figure S2. Schematic diagram of three cluster thinning treatments. (A) CT-1, one cluster per shoot thinned, 1 cluster/shoot reserved; (B) CT-2, one cluster every other shoot thinned, average 1.5 clusters/shoot reserved; (C) CT-3, not thinned, 2 clusters/shoot reserved........... 2

Supplementary tables **3**

Table S1. Meteorological data of the experimental field during the growing season (April-October) and ripening period (August-September) in 2011-2012. 3

Table S2. The concentrations of C_6 and C_9 volatiles in wines made from berries with different treatments from two farms in two vintages. 4

Table S3. The average values of the selected soils parameters in two vineyards................... 5

Table S4. Wine parameters made from berries with different treatments from two farms in two vintages. 6

Supplementary figures

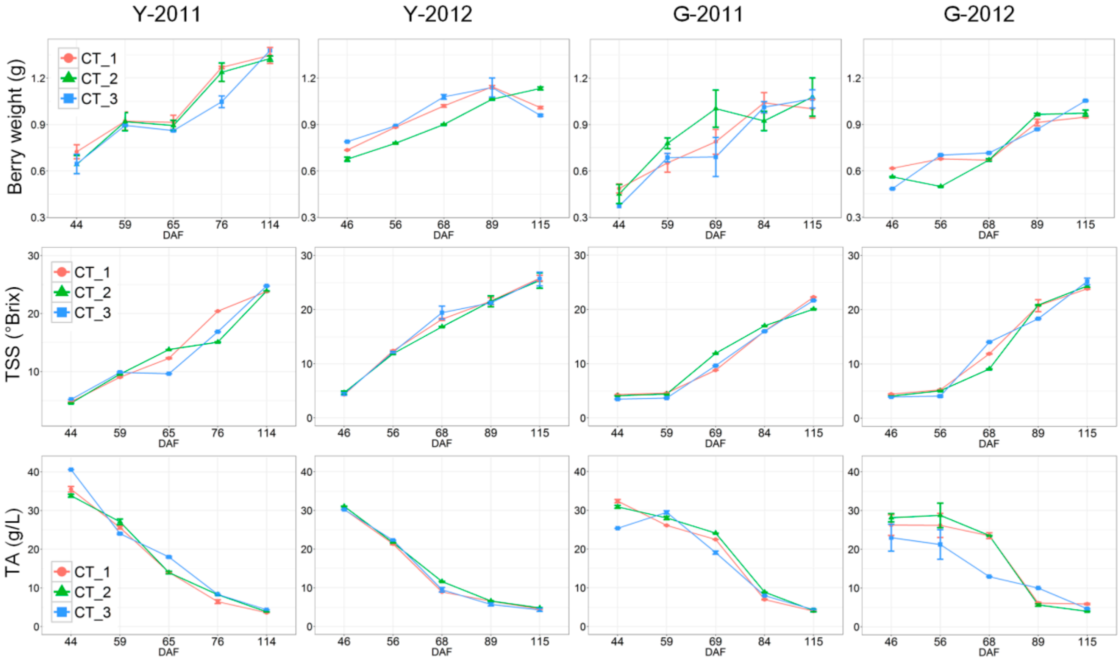

Figure S1. Effects of cluster thinning treatments on berry weight, total soluble solids (TSS) content and titratable acidity (TA) in berries of Cabernet Sauvignon during grape development in two seasons (2011-2012). CT-1, one cluster per shoot thinned, 1 cluster/shoot reserved; (B) CT-2, one cluster every other shoot thinned, average 1.5 clusters/shoot reserved; (C) CT-3, not thinned, 2 clusters/shoot reserved.

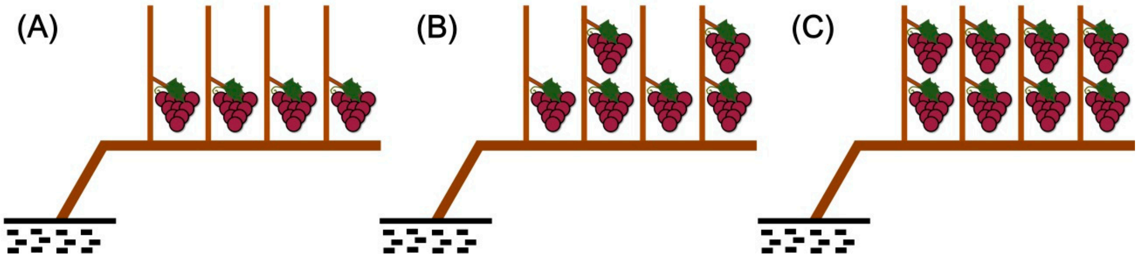

Figure S2. Schematic diagram of three cluster thinning treatments. (A) CT-1, one cluster per shoot thinned, 1 cluster/shoot reserved; (B) CT-2, one cluster every other shoot thinned, average 1.5 clusters/shoot reserved; (C) CT-3, not thinned, 2 clusters/shoot reserved.

Supplementary tables

Table S1. Meteorological data of the experimental field during the growing season (April-October) and ripening period (August-September) in 2011-2012.

Year	Mean Temperature (°C)		Growing Degree Days (°C)		Sunlight Duration (h)		Rain (mm)	
	Aug-Sep	Apr-Oct	Aug-Sep	Apr-Oct	Aug-Sep	Apr-Oct	Aug-Sep	Apr-Oct
2011	22.1	20.3	740.9	2290.6	627.4	2173.7	41.2	181.1
2012	22.3	20.8	752.9	2356.2	647.3	2235.8	29.0	103.6

Table S2. The concentrations of C_6 and C_9 volatiles in wines made from berries with different treatments from two farms in two vintages.

Wine volatiles (μg/L)	Y-farm						G-farm					
	2011			2012			2011			2012		
	CT-1	CT-2	CT-3	CT-1	CT-2	CT-3	CT-1	CT-2	CT-3	CT-1	CT-2	CT-3
Ethyl hexanoate (mg/L)	0.84±0.30a*	0.63±0.19a	0.59±0.20a	3.72±0.10a	4.58±0.15b	3.93±0.24ab	0.54±0.07a	0.75±0.03a	0.87±0.16a	4.42±0.47a	3.87±0.29a	4.54±0.11a
Hexyl acetate	16.05±6.45a	9.03±4.4a	7.72±1.18a	98.97±2.7b	21.43±0.56a	164.59±8.55c	23.67±7.44a	35.36±1.48a	44.25±8.03a	314.8±32.19a	316.98±24.05a	324.45±7.32a
Ethyl 3-hexenoate	nd**	nd	nd	2.23±1.65a	5.91±0.27a	2.3±2.05a	nd	nd	nd	6.11±0.54a	6.17±0.12a	6.66±0.04a
Propyl hexanoate	nd	nd	nd	8.58±0.21a	12.47±0.5b	9.1±0.24a	nd	nd	nd	11.79±1.47a	10.44±1.3a	14.59±0.53a
Ethyl 2-hexenoate	12.16±3.08a	6.38±1.83a	8.11±1.24a	15.52±0.24c	11.36±0.23a	12.61±0.16b	6.72±0.72a	6.56±0.25a	9.87±0.6b	15.35±1.13a	14.96±0.51a	17.13±0.51a
1-Hexanol (mg/L)	1.86±0.05a	1.38±0.17a	1.72±0.27a	0.36±0.02b	0.27±0.02a	0.31±0.01ab	1.56±0.01a	2.22±0.07b	2.41±0.09b	0.45±0.01a	0.46±0.01a	0.48±0.01a
(E)-3-Hexen-1-ol	23.36±1.21a	16.83±1.37a	23.49±4.42a	95.79±0.58c	71.74±0.65a	84.01±1.81b	22.02±1.41a	30.08±0.6b	35.89±0.1c	128.78±5.98a	137.71±4.36a	124.29±6.3a
(Z)-3-Hexen-1-ol	7.25±0.56a	7.51±0.15a	6.37±0.24a	94.17±7.56a	120.97±5.73ab	138.59±6.17b	9.05±0.38a	12.1±0.35b	11.46±0.78b	267.49±8.25a	278.41±5.67a	367.08±7.93b
(E)-2-Hexen-1-ol	6.77±9.57a	nda	nda	nd	nd	nd	nd	nd	nd	nd	nd	nd
Ethyl 8-nonenoate	1.14±1.62a	1.24±1.75a	2.57±0.12a	3.34±0.54a	3.57±0.01a	3.5±0.01a	1.52±2.14a	1.55±2.19a	2.53±1.36a	3.47±0.01a	3.47±0a	3.44±0.03a
(Z)-3-Nonen-1-ol	nd	nd	nd	0.36±0.1a	0.29±0a	0.29±0a	nd	nd	nd	0.29±0a	0.68±0.16b	0.29±0a
(E)-6-Nonen-1-ol	1.02±0.03a	0.98±0.28a	1.06±0.01a	1.38±0.02b	1.08±0.1a	1.31±0.04ab	nd	nd	nd	1.5±0.06a	1.34±0.13a	1.55±0.15a
(E,Z)-2,6-Nonadienal	nd	nd	nd	3.11±0.23b	3.67±0.05b	2.09±0.16a	nd	nd	nd	3.35±0.47a	3.04±0.07a	2.97±0.02a
Hexanoic acid (mg/L)	0.58±0.21a	0.40±0.08a	0.37±0.11a	nd	nd	nd	0.47±0.13a	0.56±0.12a	0.47±0.05a	nd	nd	nd
Esters (mg/L)	0.86±0.31a	0.65±0.20a	0.61±0.02a	3.85±0.10a	4.63±0.15b	4.12±0.24ab	0.58±0.08a	0.79±0.03a	0.93±0.02a	4.77±0.51a	4.22±0.32a	4.90±0.12a
Alcohols (mg/L)	1.90±0.06a	1.40±0.17a	1.75±0.28a	0.56±0.01b	0.47±0.02a	0.54±0.00b	1.59±0.00a	2.26±0.07b	2.46±0.09b	0.85±0.03a	0.87±0.01ab	0.97±0.03b
C_9	2.16±1.59a	2.22±2.04a	3.63±0.13a	8.19±0.68a	8.61±0.04a	7.19±0.21a	1.52±2.14a	1.55±2.19a	2.53±1.36a	8.62±0.55a	8.53±0.21a	8.26±0.16a
C_6 (mg/L)	3.34±0.58a	2.45±0.10a	2.73±0.36a	4.40±0.11a	5.10±0.17a	4.65±0.25a	2.63±0.22a	3.62±0.23b	3.86±0.21b	5.62±0.53a	5.09±0.31a	5.87±0.15a
Sum*** (mg/L)	3.35±0.58a	2.45±0.11a	2.73±0.37a	4.41±0.11a	5.11±0.17a	4.66±0.25a	2.63±0.22a	3.62±0.23b	3.86±0.21b	5.63±0.54a	5.10±0.31a	5.88±0.15a

*Different letters represent significant differences among treatments from individual farm in each vintage according to Duncan test ($P < 0.05$).

**nd means not detected. ***Sum represents the total concentration of all C_6 and C_9 volatiles.

Table S3. The average values of the selected soils parameters in two vineyards.

Vineyards	Sand (%)	pH	Organic Matter (%)	Water Content (%)	Elements (mg/kg)					
					Na	Ca	Mg	N	P	K
Y-farm	35.2	7.9	0.8	25.1	33.0	3711.4	231.1	59.1	4.7	133.2
G-farm	29.9	7.3	6.1	36.5	58.3	3798.0	367.0	79.9	23.4	104.9

Table S4. Wine parameters made from berries with different treatments from two farms in two vintages.

Treatment	Ethanol (%vol)		Residual sugar (g/L)		pH	
**Y-farm**	2011	2012	2011	2012	2011	2012
CT-1	13.70	15.20^{b}	2.90	2.68^{a}	3.99	3.88^{a}
CT-2	13.70	14.00^{a}	2.88	3.33^{b}	3.98	3.94^{b}
CT-3	14.00	15.10^{b}	2.55	2.70^{a}	3.94	3.87^{a}
Significance	ns	*	ns	*	ns	*
**G-farm**	2011	2012	2011	2012	2011	2012
CT-1	13.10	13.30	3.30	3.61	4.00^{b}	3.76^{b}
CT-2	12.30	13.70	3.11	3.90	3.93^{a}	3.76^{b}
CT-3	12.70	13.20	3.23	3.80	3.93^{a}	3.75^{a}
Significance	ns	ns	ns	ns	*	*

Different letters represent significant differences among treatments in each vintage according to Duncan test. *: significant at 0.05 level. Cluster thinning treatments: CT-1, one cluster per shoot thinned, 1 cluster/shoot reserved; CT-2, one cluster every other shoot thinned, average 1.5 clusters/shoot reserved; CT-3, not thinned, 2 clusters/shoot reserve.
